# The functional neuroanatomy of musical memory in Alzheimer's disease

**DOI:** 10.1016/j.cortex.2019.02.003

**Published:** 2019-06

**Authors:** Catherine F. Slattery, Jennifer L. Agustus, Ross W. Paterson, Oliver McCallion, Alexander J.M. Foulkes, Kirsty Macpherson, Amelia M. Carton, Emma Harding, Hannah L. Golden, Kankamol Jaisin, Catherine J. Mummery, Jonathan M. Schott, Jason D. Warren

**Affiliations:** Dementia Research Centre, UCL Queen Square Institute of Neurology, University College London, London, United Kingdom

**Keywords:** Alzheimer's disease, Posterior cortical atrophy, Dementia, Music, Memory, fMRI

## Abstract

**Background:**

Memory for music has attracted much recent interest in Alzheimer's disease but the underlying brain mechanisms have not been defined in patients directly. Here we addressed this issue in an Alzheimer's disease cohort using activation fMRI of two core musical memory systems.

**Methods:**

We studied 34 patients with younger onset Alzheimer's disease led either by episodic memory decline (typical Alzheimer's disease) or by visuospatial impairment (posterior cortical atrophy) in relation to 19 age-matched healthy individuals. We designed a novel fMRI paradigm based on passive listening to melodies that were either previously familiar or unfamiliar (musical semantic memory) and either presented singly or repeated (incidental musical episodic memory).

**Results:**

Both syndromic groups showed significant functional neuroanatomical alterations relative to the healthy control group. For musical semantic memory, disease-associated activation group differences were localised to right inferior frontal cortex (reduced activation in the group with memory-led Alzheimer's disease); while for incidental musical episodic memory, disease-associated activation group differences were localised to precuneus and posterior cingulate cortex (abnormally enhanced activation in the syndromic groups). In post-scan behavioural testing, both patient groups had a deficit of musical episodic memory relative to healthy controls whereas musical semantic memory was unimpaired.

**Conclusions:**

Our findings define functional neuroanatomical substrates for the differential involvement of musical semantic and incidental episodic memory in major phenotypes of Alzheimer's disease. The complex dynamic profile of brain activation group differences observed suggests that musical memory may be an informative probe of neural network function in Alzheimer's disease. These findings may guide the development of future musical interventions in dementia.

## List of abbreviations

AD: Alzheimer's disease
dB: decibels
CSF: cerebrospinal fluid
DARTEL: Diffeomorphic Anatomical Registration Through Exponentiated Lie algebra
DSMT: Digit Symbol Modalities Test
F: female
FoV: field of view
fMRI: functional magnetic resonance imaging
GE-EPI: gradient-echo echo-planar image
GDA: Graded Difficulty Arithmetic
GDST: Graded Difficulty Spelling Test
GNT: Graded Naming Test
GRAPPA: GeneRalized Autocalibrating Partial Parallel Acquisition
FEW: family wise error
IQ: intelligence quotient
M: male
mAD: memory-led Alzheimer's disease
mm: millimeters
MMSE: mini-mental state examination
MRI: magnetic brain imaging
Ms: msec
N: number
NART: National Adult Reading Test
P: probability
PCA: posterior cortical atrophy
RMT: Recognition Memory Test
SD: standard deviation
SPM: statistical parametric mapping
T: tesla
TE: echo time
TR: repetition time
VBM: voxel based morphometry
VOSP: Visual Object and Spatial Perception battery
WASI: Wechsler Abbreviated Scale of Intelligence
WMS-R: Wechsler Memory Scale Revised

## 1 Introduction

Despite considerable interest, the neural mechanisms underlying musical memory in Alzheimer's disease (AD) remain contentious. Music engages the separable cognitive systems mediating procedural memory (playing an instrument), semantic memory (recognition of musical objects, such as familiar tunes) and episodic memory (encoding and recollection of specific musical events) (Baird and Samson, 2015, Omar et al., 2012). These musical memory systems are likely to be differentially vulnerable to the effects of AD (Baird and Samson, 2015, Groussard et al., 2013, Jacobsen et al., 2015, Omar et al., 2012). The balance of evidence suggests that episodic memory for music becomes impaired early in the course of AD while effects on musical semantic and procedural memory are more variable and may become more evident with advancing disease (Baird and Samson, 2015, Groussard et al., 2013, Omar et al., 2010, Vanstone et al., 2012), mirroring memory functions in non-musical domains (Warren, Fletcher, & Golden, 2012) and providing a potential mechanism for familiar music to ‘unlock’ autobiographical memories and other cognitive capacities in AD (Cuddy, Sikka, & Vanstone, 2015). Functional neuroanatomical work in the healthy brain has identified separable, distributed, bi-hemispheric cerebral networks that support these musical memory systems. Musical semantic memory has been shown to engage anterior temporal, inferior and supero-medial prefrontal cortices (Groussard, La Joie et al., 2010, Groussard, Rauchs et al., 2010, Jacobsen et al., 2015, Platel et al., 2003, Sikka et al., 2015) while musical episodic memory engages precuneus, posterior cingulate, hippocampus and other mesial temporal lobe structures (Burunat et al., 2014, Platel et al., 2003, Watanabe et al., 2008). The processing of unfamiliarity (novelty) in music and other sensory stimuli activates a distributed network of brain areas overlapping those implicated in musical semantic and episodic memory, including mesial temporal lobes and temporoparietal, inferior frontal, insula and anterior cingulate cortices (Downar et al., 2002, Herdener et al., 2010, Hunkin et al., 2002). This emerging picture of the functional neuroanatomy of musical memory aligns with neuroanatomical and neuropathological studies of AD pathogenesis. Substantial evidence has implicated a core neural network as the key target of pathogenic protein spread in AD (Buckner et al., 2008, Seeley et al., 2009, Warren et al., 2012, Lehmann et al., 2010): this ‘default mode’ network links medial temporal lobe structures to lateral temporo-parietal and medial prefrontal regions via a ‘hub’ zone in postero-medial cortex (posterior cingulate and precuneus). In addition to mediating stimulus-independent thought (Fox, Snyder, Vincent, Corbetta, Van Essen & Raichle, 2005) the default mode network plays an active role in coordinating brain activity during various other cognitive operations, including the analysis of auditory scenes and patterns (Leech and Sharp, 2014, Zundorf et al., 2013). Involvement of this network underpins clinical deficits in the major AD variant phenotypes of clinically typical AD, led by memory decline (mAD) and posterior cortical atrophy (PCA), dominated by early visuoperceptual and spatial impairment due to relatively selective involvement of parieto-temporal areas (Crutch et al., 2012, Golden, Augustus et al., 2015, Golden et al., 2016, Goll et al., 2012, Tang-Wai et al., 2004, Warren et al., 2012). In both syndromes, impaired processing of complex auditory stimuli has been linked specifically to dysfunction and atrophy of the postero-medial cortical hub region (Crutch et al., 2012, Golden, Augustus et al., 2015, Golden, Nicholas et al., 2015, Golden et al., 2016, Goll et al., 2012, Warren et al., 2012). It has been proposed that preservation of musical memory (more particularly, musical semantic memory) in AD might be attributable to relative sparing of medial prefrontal cortices implicated in mediating musical familiarity in healthy listeners (Jacobsen et al., 2015): the crucial next step is to measure changes in musical memory processing directly in patients with AD, and to dissect apart disease effects on particular core musical memory systems.

Here we addressed this issue using activation fMRI in a cohort of patients representing the canonical syndromes of mAD and PCA, relative to healthy age-matched individuals. We chose to study younger patients whose functional neuroanatomy is likely to provide a ‘purer’ index of the effects of AD pathology, not confounded by concurrent cerebrovascular and other comorbidities. We designed a simple paradigm equipped to capture disease-associated changes in the core semantic and episodic dimensions of musical memory that might be relevant to any listener, including those without specific musical training. In everyday music listening, we are generally not called upon to analyse melodies explicitly but the sense that a tune is familiar, that musical motifs are repeating or that new material is being presented are common experiences that contribute importantly to the appreciation of music even among musically naïve listeners. These listening experiences are in turn likely to depend on semantic and episodic musical memory systems: prior familiarity with a melody engages musical semantic memory while the incidental detection of repeating motifs engages (incidental) episodic memory for music. In our fMRI paradigm, we manipulated these factors of prior familiarity and repetition orthogonally in a stimulus set comprising short musical melodies. Although familiarity decisions on melodies have been widely used as a cipher for musical semantic memory in previous studies (Omar et al., 2012), familiarity does not, of course, equate to detailed semantic knowledge of a musical piece; nor does incidental memory for music equate to explicit episodic recall. Our objective here was not to delineate fully the brain systems that mediate musical semantic and episodic memory, but rather to probe these systems using a paradigm relevant to listeners potentially varying widely in musical expertise. In addition, we wished to avoid the requirement for an overt task during scanning, since task and related performance monitoring effects can be particularly problematic in fMRI studies of cognitively impaired individuals. In order to provide a behavioural reference for the neuroanatomical changes observed, all participants completed tasks assessing musical semantic and episodic memory following the scanning session.

Based on a synthesis of behavioural and neuroanatomical evidence (Baird and Samson, 2015, Burunat et al., 2014, Groussard, Rauchs et al., 2010, Groussard et al., 2013, Jacobsen et al., 2015, Omar et al., 2012, Platel et al., 2003, Sikka et al., 2015, Vanstone et al., 2012, Warren et al., 2012, Watanabe et al., 2008), we hypothesised that AD and PCA would be associated with a similar profile of abnormal activation of postero-medial cortices during incidental episodic processing of repeated melodies relative to healthy individuals, whereas these syndromes would show divergent activation profiles during semantic processing of prior melody familiarity, due to sparing of more anterior cortical regions in PCA relative to mAD.

## 2 Materials and methods

### 2.1 Participants

Thirty-four consecutive patients (mean age 60.9 years; 20 female) fulfilling consensus criteria for AD (McKhann et al., 2011) and 19 age-matched healthy individuals (60.5 years, 10 female) with no history of neurological or psychiatric illness participated in the study. We targeted younger patients with AD (onset before age 65 years) as the phenotype in these patients is less likely to be confounded by vascular or other comorbidities. Twenty-four patients presented with an amnestic clinical syndrome of mAD (Dubois et al., 2014) and 10 patients presented with a syndrome meeting research criteria for PCA (Tang-Wai et al., 2004). The clinical syndromic diagnosis was corroborated by a general neuropsychological assessment in all cases; on volumetric brain MRI, all patients had a compatible profile of regional atrophy with no significant associated burden of cerebrovascular disease. No participant had a history of hearing loss or congenital amusia. Demographic, clinical and neuropsychological details for all participant groups are summarised in Table 1. CSF examination was undertaken in 30 patients (23/24 with mAD, 7/10 with PCA), all of whom had a profile of neurodegeneration protein markers (tau and amyloid_1-42_) consistent with likely underlying AD pathology (Weston et al., 2015). At the time of participation, 32 patients were receiving symptomatic treatment with an acetylcholinesterase inhibitor (two were also taking memantine), one patient was taking memantine without a cholinesterase inhibitor and the remaining patient was taking no symptomatic treatment.

**Table 1:** Demographic, clinical and behavioural characteristics of participant groups.

Characteristic	Healthy controls n = 19	mAD n = 24	PCA n = 10
**General**			
Gender (M:F)	9:10	12:12	2:8
Age (years)	60.5 (6.0)	60.3 (4.4)	62.1 (5.6)
Handedness (L:R)	3:16	1:23	1:9
Education (years)	17.1 (3.1)	15.5 (2.9)	15.0 (2.9)
Musical training (years)	2.7 (5.2)	3.8 (5.3)	1.2 (2.1)
Music listening (hours/week	9.4 (10.5)	6.8 (7.4)	5.9 (8.1)
MMSE (/30)	29.5 (.7)	19.7* (3.7)	22.9* (4.3)
Age at onset (years)	NA	55.2 (3.9)	55.8 (4.3)
Symptom duration (years)	NA	5.1 (2.6)	6.3 (3.3)
CSF examination	NA	23^§^	7^§^
**Neuropsychological assessment**			
*Episodic memory*			
RMT words (short,/25)	24.5 (.8)	**16.8**†* (2.5)	20.0†* (4.0)
RMT faces (short,/25)	24.5 (1.0)	20.0† (4.6)	18.3† (4.7)
*Executive skills*			
WASI matrices (/35)	26.9 (2.3)	9.0 †*(6.6)	**3.6** †*(3.8)
WMS-R digit span forward (/12)	8.9 (1.8)	5.8† (2.1)	6.2† (3.0)
WMS-R digit span backwards (/12)	7.8 (1.6)	3.7† (1.6)	3.9† (2.7)
DSMT (/93)	54.6 (9.1)	**13.4** † **(11.9)**	**5.8** † **(8.7)**
A cancellation	20.7 (5.1)	**50.2**†* (20.5)	**74.5**†* (18.0)
*Verbal skills*			
NART (/50)	40.3 (5.1)	30.6† (10.3)	28.4† (12.4)
WASI vocabulary (/80)	69.7 (7.5)	53.0† (17.4)	55.5† (21.7)
GNT (/30)	25.7 (2.7)	**14.1**† **(9.3)**	17.6† (7.1)
*Literacy and numeracy skills*			
GDST (/30)	27.4 (3.0)	16.1† (8.6)	13.5† (8.1)
GDA (/24)	13.7 (6.7)	**1.8** † **(2.8)**	**1.5**† **(2.0)**
*Visuoperceptual skills*			
VOSP object decision (/20)	18.2 (1.5)	15.6†* (3.0)	**10.4**†* (4.5)
VOSP shape detection (/20)	19.4 (.8)	**18.3**†* (1.4)	**16.2**†* (2.4)
VOSP fragmented letters (/20)	19.4 (.7)	**12.3**†* (7.1)	**5.3**†* (5.6)
VOSP dot counting (/10)	9.9 (.3)	8.1†* (2.8)	**4.9**†* (3.0)
**Post-scan musical tasks**			
Melody familiarity judgement (d’)	2.8 (.7)	2.8 (.8)	2.7 (.8)
Melody episodic memory^#^ (d’)	1.6 (.8)	.8† (.7)	.9† (.6)

Mean (standard deviation) values are presented unless otherwise indicated. Raw data are shown for neuropsychological tests (maximum scores in parentheses). Bold indicates patient performance was significantly impaired (<5th percentile) relative to age matched published norms; †significant difference between patient group and healthy controls (*p* < .05), *significant difference between patient syndromic groups (*p* < .05); #18 controls, 14 mAD, eight PCA patients completed this test; §profile of neurodegeneration markers consistent with AD pathology in all cases; CSF, cerebrospinal fluid; DSMT, Digit Symbol Modalities Test (Smith and Helmuth, 1986); F, female; GDA, Graded Difficulty Arithmetic (Jackson & Warrington, 1986); GDST, Graded Difficulty Spelling Test (Baxter & Warrington, 1994); GNT, Graded naming test (McKenna, Warrington, NFER-Nelson, & Windsor, 1983); IQ, intelligence quotient; M, male; MMSE, Mini Mental State Examination (Folstein, Folstein, & McHugh, 1975); n, number; NA, not applicable; NART, National Adult Reading Test (Nelson, 1982); P, probability; PCA, patient group with posterior cortical atrophy; RMT, Recognition Memory Test (Warrington, 1984); verbal fluency (Gladsjo, Schuman, Evans, Peavy, Miller & Heaton, 1999); mAD, patient group with typical amnestic presentation of Alzheimer's disease; VOSP, Visual Object and Spatial Perception battery (Warrington & James, 1991); WASI, Wechsler Abbreviated Scale of Intelligence (Wechsler, 1999); WMS-R, Wechsler Memory Scale Revised (Wechsler, 1987).

### 2.2 Assessment of musical background and peripheral hearing function

All participants completed a questionnaire detailing their prior musical training and current music listening (Hailstone, Omar, Henley, Frost, Kenward & Warren, 2009). In order to assess peripheral hearing function, all participants had pure-tone audiometry using a procedure adapted from a commercial screening audiometry software package (http://www.digital-recordings.com/audiocd/audio.html). The test was administered via headphones from a laptop in a quiet room. Five frequency levels (500, 1000, 2000, 3000, 4000 Hz) were assessed: at each frequency participants were presented with an intermittent tone that slowly and linearly increased in intensity. Participants were instructed to indicate as soon as they were sure they could detect the tone; this response time was measured and stored for offline analysis.

### 2.3 Experimental stimuli and protocol

Two musical stimulus subsets were created, based respectively on previously familiar melodies (widely-known tunes that would be recognised based on long-term, general musical experience rather than specific autobiographical recall) and previously unfamiliar melodies (melodies created de novo for the experiment). During scanning, particular melodies from each set were either presented once only or twice, thereby varying the frequency of particular musical events or episodes over the experimental session. This yielded four stimulus conditions: familiar melodies, each presented once; unfamiliar melodies, each presented once; familiar melodies, each presented twice; unfamiliar melodies, each presented twice. This experimental design allowed construction of key contrasts to assess musical semantic memory (previously familiar > unfamiliar melodies), musical novelty (the ‘reverse’ contrast, previously unfamiliar > familiar melodies), musical episodic memory [repeat (second) > first presentation of repeated melodies] and musical encoding (the ‘reverse’ contrast, first > repeat presentation of repeated melodies).

Musical stimuli were synthesised in MatlabR2012a^®^ as digital wavefiles (sampling rate 44.1 kHz) and comprised sequences of harmonic complexes (notes) with defined fundamental pitch and fixed inter-note gap (6 msec). The total length of each stimulus sequence was fixed at eight seconds and mean sound intensity was fixed across stimuli. Familiar melodies comprised excerpts from popular classical instrumental tunes widely known among older British individuals (details in Table S1 in Supplementary Material online), selected based on a pilot survey; the tune excerpts selected were classified as highly familiar by ≥ 80% of healthy older British participants (n = 5, all >50 years, none of whom participated subsequently in the fMRI study). Non-vocal tunes were used to minimise implicit processing of verbal (song lyric) associations. Unfamiliar melodies were created by re-distributing the notes from each familiar melody to create a novel musical sequence with equivalent temporal and pitch interval structure. Repeated melodies (half previously familiar, half unfamiliar) were distributed such that repeats were separated by two intervening melodies (stimulus trials) which [in the ‘sparse’ (long TR) acquisition protocol used here] corresponded to an interval of approximately 33 sec between repetitions of a given note sequence. This design was intended to maximise any effect from melody repetition while minimising musical short-term sensory trace memory or working memory processing. A silent ‘rest’ condition was also included to provide a low-level baseline for assessing the effect of auditory stimulation.

The complete stimulus set (144 trials) comprised 96 unique melodies (48 familiar, 48 unfamiliar) plus 48 repeat-presentation trials (24 familiar melodies, 24 unfamiliar melodies). The stimuli were delivered binaurally from a laptop using electrodynamic headphones (MR Confon GmbH, Magdeburg) at a comfortable listening level (approximately 70 dB). The presentation order of familiar and unfamiliar melody trials was randomised. In-house routines in Python (http://www.python.org) were used to integrate stimulus delivery with the scanner controls. Participants were instructed to listen to the sound stimuli; there was no output task and no participant responses were collected during the scanning session.

### 2.4 Brain image acquisition

MRI data were acquired on a 3T TIM Trio whole-body MRI scanner (Siemens Healthcare, Erlangen, Germany). 144 gradient-echo echo-planar image (GE-EPI) volumes were acquired using a 12-channel RF receive head and body transmit coil in sparse (TR 11.3 sec) acquisition mode (to reduce any interaction between scanner acoustic noise and auditory stimulus presentations). Each EPI volume comprised 48 oblique transverse slices with slice thickness 2 mm, inter-slice gap 1 mm and 2 × 2 mm in-plane resolution (TR/TE = 11340/30 msec; echo spacing = .69 msec; matrix size = 96 × 96 pixels; FoV = 192 × 192 mm, GRAPPA factor 2 in anterior-posterior phase encoding direction). The initial two brain volumes were discarded to allow for equilibrium of longitudinal T1 magnetization. B0 field-maps were acquired using two gradient echo sequences (TR = 688 msec; TE1/TE2 = 4.92/7.38 msec, 3 × 3 x 3 mm resolution, no inter-slice gap; matrix size = 80 × 80pixels; FoV = 192 × 192 mm; phase encoding anterior-posterior) to allow correction of field inhomogeneity.

Volumetric structural brain MR images were also obtained in each participant. A sagittal 3-D magnetization prepared rapid gradient echo T1-weighted volumetric MRI (TR/TE = 2200/2.9 msec, dimensions 256 × 256 × 208, voxel size 1.1 × 1.1 × 1.1 mm) was acquired on the same 3.0T Siemens scanner using a 32-channel phased-array head coil.

### 2.5 Post-scan behavioural assessment

Immediately after the scanning session two behavioural tests based on the fMRI conditions were administered, in order to assess each participant's ability to process relevant dimensions of musical memory. To assess musical semantic memory, 24 (12 familiar, 12 unfamiliar) melodies from the set used during scanning were presented in randomised order and the task on each trial was to decide whether the tune was familiar or unfamiliar. The second test was designed to assess musical episodic recognition memory using novel musical stimuli. The participant was first asked to assess the pleasantness of three probe melodies (not previously presented during scanning) and then, after a delay of 60 sec, to identify these melodies among nine foil melodies (the task on each trial was to decide whether or not the melody had been presented earlier); this same procedure was repeated for a second set of probes and foils. Participant responses were given verbally and recorded by the researcher. No feedback was given during either test and no time limits were imposed. Participant responses were recorded for off-line analysis.

### 2.6 Data analyses

#### 2.6.1 Demographic and behavioural data

All statistical analyses were conducted using Stata v12^®^. Demographic characteristics and musical experience were compared between the control and patient groups using two sample *t*-tests and Wilcoxon rank sum; gender differences were assessed using a Pearson's chi-square test. Neuropsychological data were compared between study groups using Wilcoxon rank sum tests. Tone detection thresholds on audiometry screening and musical familiarity and repetition judgement results were analysed using linear regression models with clustered, robust standard error.

Performance data for the post-scan musical memory tasks were analysed using signal detection theory. Hit rate and false alarm rates were calculated and combined to create sensitivity measure d-prime [Z (Hit rate)–Z (False alarm rate)]. Two sample *t*-tests were used to compare d-prime values between participant groups on each task.

A threshold *p* < .05 was accepted as the criterion of statistical significance for all tests.

#### 2.6.2 Voxel-based morphometry data

Structural brain MR images were compared between patient and control groups in a VBM analysis. Normalisation, segmentation and modulation of grey and white matter images were performed using default parameter settings in statistical parametric mapping software (SPM8; http://www.fil.ion.ucl.ac.uk/spm), with a Gaussian smoothing kernel of 6 mm full-width-at-half-maximum. To help protect against voxel drop-out because of potentially marked local regional atrophy in particular scans, a customised explicit brain mask was derived by maximising the correlation between the binary mask and the average of the images to be analysed (Ridgway, Omar, Ourselin, Hill, Warren & Fox, 2009). Regional grey matter volume was compared between patient and control groups and between syndromic groups using voxel-wise two sample *t*-tests, including covariates of age, gender and total intracranial volume. Statistical parametric maps of grey matter atrophy were thresholded at peak voxel level *p* < .05 after family-wise-error (FWE) correction for multiple comparisons over the whole brain volume.

#### 2.6.3 Functional MRI data

Data from the fMRI experiment were pre-processed using SPM8. Scans for each participant were realigned using the first image as a reference, and unwarped incorporating field-map distortion information. DARTEL processing was used to spatially normalise individual scans to a group mean template image in MNI space. Normalised images were smoothed using a Gaussian smoothing kernel 6 mm full-width at half-maximum.

Pre-processed images were entered into a first-level design matrix modelling each experimental condition as a separate regressor with boxcars of one-TR duration convolved with the canonical haemodynamic response function and six head movement regressors derived from the realignment process. First-level contrast images were generated for effects of musical semantic memory (familiar > unfamiliar melody conditions), musical novelty (unfamiliar > familiar melody conditions), incidental musical episodic memory (repeat > first presentation of repeated melodies) and musical encoding (first > repeat presentation of repeated melodies). Contrast images for each participant were entered into a second-level random-effects analysis using *t*-tests to examine within- and between-group effects.

Contrasts were assessed at peak voxel level within small anatomical volumes of interest, as specified by our prior hypotheses based on previous functional neuroanatomical work in the healthy brain (Burunat et al., 2014, Downar et al., 2002, Groussard, La Joie et al., 2010, Groussard, Rauchs et al., 2010, Hunkin et al., 2002, Jacobsen et al., 2015, Platel et al., 2003, Sikka et al., 2015, Strange et al., 2005, Watanabe et al., 2008). Relevant small volumes were derived from the Harvard–Oxford Brain Atlas (Desikan et al., 2006) in FSL view (Jenkinson, Beckmann, Behrens, Woolrich & Smith, 2012) (Figure S1 in Supplementary Material on-line). These regions comprised an anterior peri-Sylvian region (combining inferior frontal gyrus, frontal operculum and anterior temporal cortex) and supplementary motor cortex, for the contrast assessing musical semantic memory; a region comprising posterior cingulate cortex, precuneus and hippocampi, for the contrasts assessing musical episodic memory and encoding; and the combination of these regions for the contrast assessing musical novelty (unfamiliarity) processing. A statistical threshold *p* < .05 after family-wise error (FWE) correction for multiple comparisons (*p*_FWE_ < .05) over the pre-specified region of interest was used in assessing all contrasts. For each contrast of interest showing a significant difference between patients and healthy controls, the voxel peak effect size (beta estimate value) was extracted for correlation with post-scan behavioural test performance.

## 3 Results

### 3.1 General characteristics of participant groups

Demographic, clinical and neuropsychological data for participant groups are summarised in Table 1. Patients and controls did not differ in age, gender, years of education, years of musical training or current musical listening. The patient groups did not differ significantly in symptom duration, but the mAD group had lower mean Mini-Mental State Examination score than the PCA group (*p* = .04). These syndromic groups showed the anticipated profiles of multi-domain cognitive impairment: relative to published norms, patients with mAD had deficits of verbal episodic memory, naming, arithmetic, visual processing and executive function while patients with PCA had markedly impaired visuoperceptual and visuospatial skills but relatively preserved episodic memory and verbal skills; comparing syndromic groups, the mAD group had significantly worse verbal episodic memory performance than the PCA group, and the PCA group had significantly worse visuoperceptual skills than the mAD group.

There were no significant effects of group membership on tone detection thresholds across frequencies (F_(2,53)_ = .59, *p* = .56).

### 3.2 Post-scan behavioural data

Group performance data for the post scan behavioural tests are summarised in Table 1. Both patient groups performed significantly worse than the control group on the musical episodic memory task (mAD *p* = .005, PCA *p* = .03) but had unimpaired musical semantic memory (mAD *p* = .7, PCA *p* = .8). There were no significant performance differences between the patient groups. Performance on the musical episodic memory test was correlated with performance on the musical semantic memory test (r = .5, *p* = .04) across the patient cohort. There were no significant correlations between any musical memory task and standard neuropsychological measures of episodic memory [Recognition Memory Test for Words and Faces (Warrington, 1984), all *p* > .05].

### 3.3 Structural neuroanatomical data

**Fig. 1:**
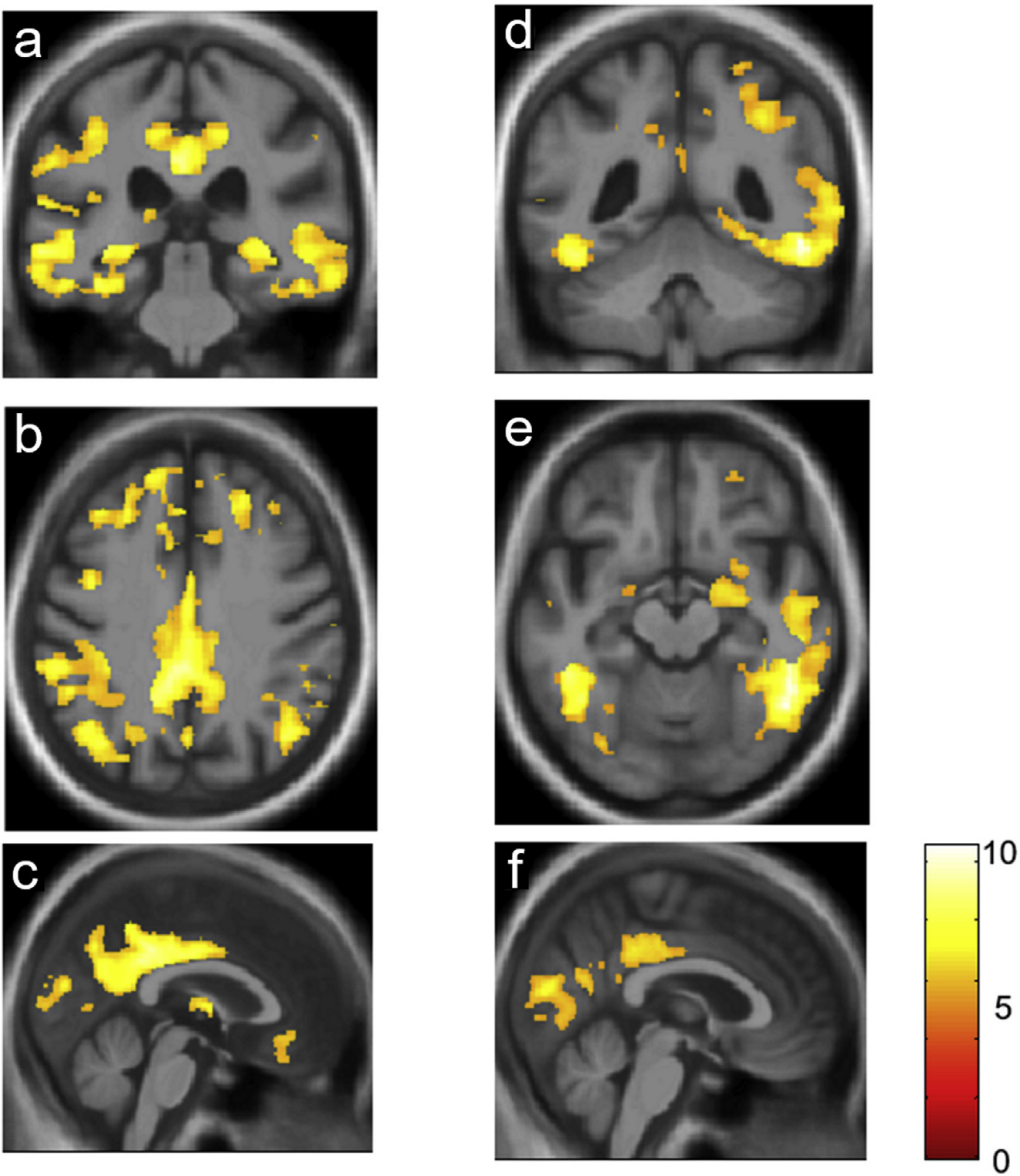
Regional grey matter atrophy profiles in the patient groups. Results of the voxel-based morphometry analysis showing statistical parametric maps (SPMs) of regional grey matter atrophy in the patient group with a memory-led syndrome of Alzheimer's disease (panels **a**, **b**, **c**) and the patient group with a syndrome of posterior cortical atrophy (panels **d**, **e**, **f**) relative to the healthy control group. SPMs are rendered on representative coronal (**a**,**d**), axial (**b**,**e**) and sagittal (**c**,**f**) sections of the group mean T1-weighted MR brain image in MNI space, thresholded at *p* < .05 after family-wise-error correction for multiple voxel-wise comparisons over the whole brain volume. The colour bar codes voxel-wise t-scores of grey matter change across the patient cohort. Planes of sections have the following MNI coordinates (mm): **a**, y = −31; **b**, z = 39; **c**, x = 0; **d**, y = −49; **e**, z = −14; **f**, x = 3. The right cerebral hemisphere is displayed on the right in coronal and axial sections.

The mAD and PCA groups showed the anticipated profiles of grey matter atrophy relative to the healthy control group (Fig. 1). The mAD group showed widespread atrophy involving the hippocampi, temporo-parietal and postero-medial cortices, also extending to involve prefrontal cortices; while the PCA group showed relatively selective posterior atrophy preferentially affecting the parietal and occipital lobes. There were no significant differences in grey matter atrophy profiles when the syndromic groups were directly compared.

### 3.4 Functional neuroanatomical data

**Fig. 2:**
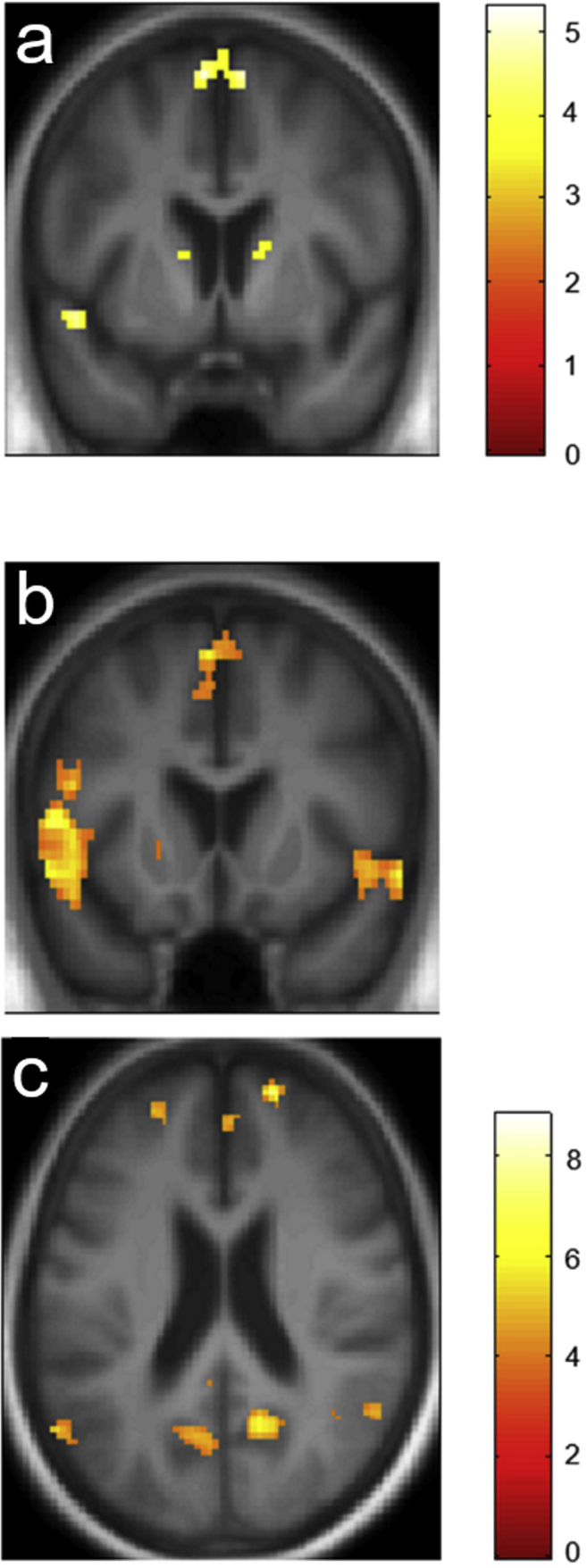
Functional neuroanatomy of musical memory: within-group correlates for patients and healthy controls. Statistical parametric maps (SPMs) show all significant regional brain activations for musical memory contrasts within participant groups; significant activations were demonstrated in the patient group with memory-led Alzheimer's disease (panel **a**) and in the healthy control group (panels **b**,**c**). Contrasts forming SPMs were as follows: **a,b** previously familiar [widely-known] > unfamiliar [newly-created] melody conditions (musical semantic memory); **c**, unfamiliar > previously familiar melody conditions (musical novelty). SPMs are rendered on coronal (**a,b**) and axial (**c**) sections of the group mean T1-weighted structural MR brain image, thresholded for display purposes at *p* < .001 uncorrected over the whole brain volume; sections have been selected to show activations significant at *p* < .05 after family-wise-error correction for multiple voxel-wise comparisons within the pre-specified small anatomical volumes of interest (see Table 2). Colour bars alongside panels **a** and **c** code voxel-wise activation t-scores in the AD group and the healthy control group. Planes of sections have the following MNI coordinates (mm): **a**, y = 6; **b**, y = 16; **c**, z = 22. The right cerebral hemisphere is displayed on the right in all panels.

**Fig. 3:**
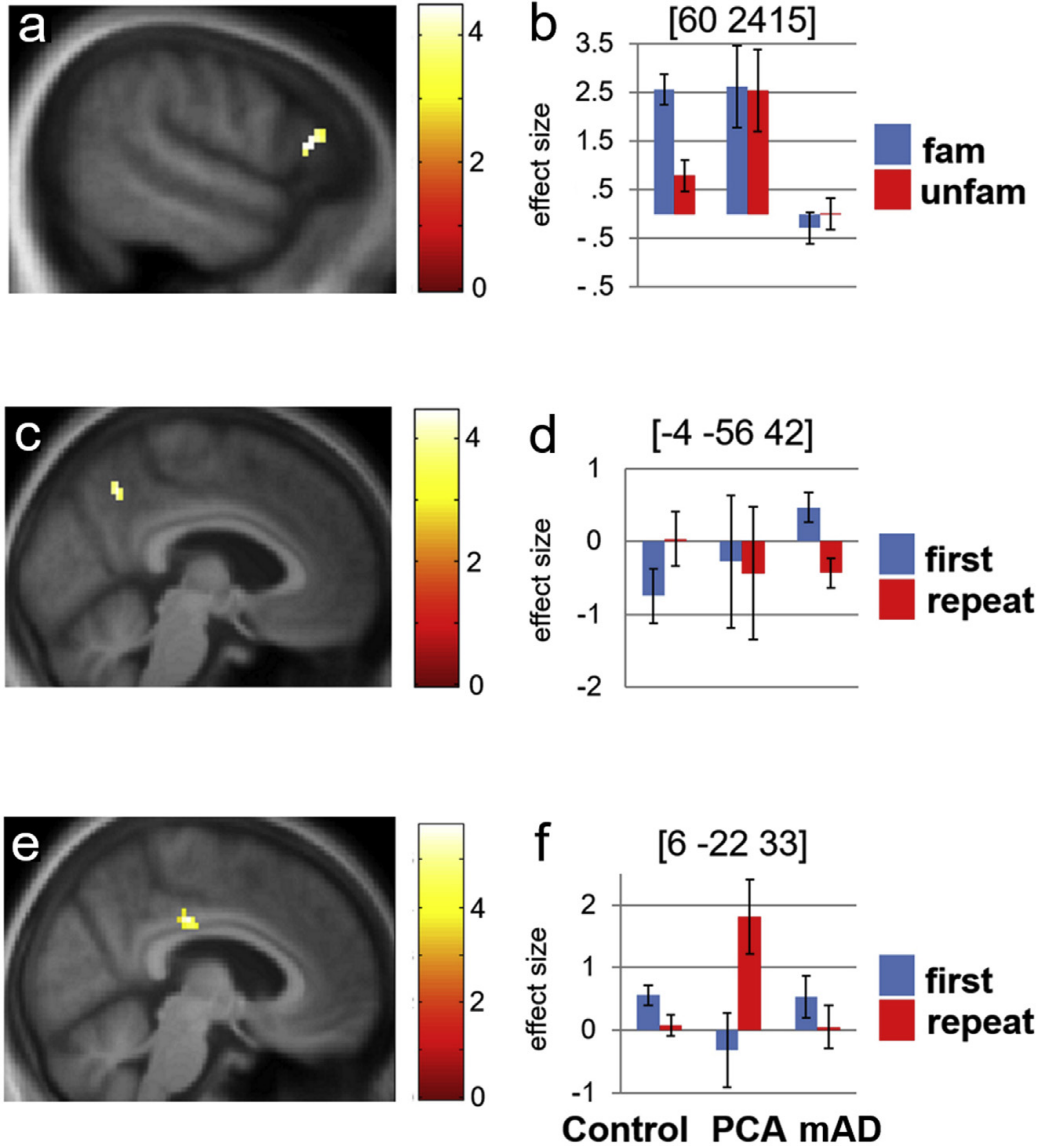
Functional neuroanatomy of musical memory: patients compared with healthy controls. Panels **a** and **b** compare the mAD and healthy control groups in the musical semantic memory contrast (previously familiar > unfamiliar melody conditions); panels **c** and **d** compare the mAD and healthy control groups in the musical episodic memory contrast (repeat > first melody presentations); panels **e** and **f** compare the PCA and healthy control groups in the musical episodic memory contrast (see also text and Table 2). Statistical parametric maps (SPMs) of significant differences in regional brain activation between groups are presented in panels **a**, **c**, **e**; plots of peak voxel condition effect size (mean beta parameter estimate ± standard error, with MNI coordinates of corresponding local maxima) are presented in panels **b**, **d**, and **f**. SPMs are rendered on sagittal sections of the group mean T1-weighted structural MR brain image, thresholded for display purposes at *p* < .001 uncorrected over the whole brain volume; activations shown were significant at *p* < .05 after family-wise-error correction for multiple voxel-wise comparisons over the anatomical small volume of interest. Colour bars alongside panels **a**, **c** and **e** code voxel-wise activation t-values for each comparison. **Control,** healthy control group; **fam**, previously familiar melodies condition; **first**, first presentation of repeated melodies; **PCA**, patient group with a syndrome of posterior cortical atrophy; **repeat**, second presentation of repeated melodies; **mAD**, patient group with a syndrome of memory-led Alzheimer's disease; **unfam**, unfamiliar melodies condition.

Regional activation profiles for the musical contrasts of interest are summarised in Table 2. Statistical parametric maps are presented in Fig. 2 (within participant groups) and Fig. 3 (comparing patients and healthy controls). All contrasts are thresholded at *p* < .05_FWE_ after correction for multiple voxel-wise comparisons within the pre-specified anatomical small volume of interest.

**Table 2:** Summary of functional neuroanatomical data within and between participant groups.

Group	Contrast	Region	Side	Cluster (voxels)	Peak (mm)			t-value	p-value
					x	y	z		
Healthy controls	Familiar > unfamiliar	Supplementary motor	R	85	6	2	66	8.83	<.001
		Supplementary motor	L	30	0	8	63	4.42	.05
		Anterior superior temporal cortex	L	312	−54	14	6	6.78	.003
		Anterior superior temporal cortex	R	90	58	14	−9	6.44	.004
		Inferior frontal gyrus	R	55	58	26	18	6.28	.006
	Unfamiliar > familiar	Precuneus	R	95	14	−58	18	7.43	.006
mAD	Familiar > unfamiliar	Supplementary motor	L	49	−6	6	63	5.03	.006
		Supplementary motor	R	25	8	6	63	4.89	.008
		Anterior superior temporal cortex	L	38	−52	10	−12	4.91	.033
Control > mAD	Familiar > unfamiliar	Inferior frontal gyrus	R	13	60	24	15	4.46	.032
mAD > Control	First > repeat presentation	Precuneus	L	13	−4	−56	42	4.21	.049
PCA > Control	Repeat > first presentation	Posterior cingulate cortex	R	20	6	−22	33	5.73	.002

Cerebral activations significant at peak voxel threshold *p* < .05_FWE_ after correction for multiple voxel-wise comparisons within the pre-specified anatomical volume of interest are shown, for clusters >10 voxels; coordinates of local maxima are in MNI space. Significant within-group contrasts are presented above and significant between-group comparisons below (the contrasts between familiar [widely-known] and unfamiliar [newly-created] melody conditions refer to familiarity prior to scanning, i.e., musical semantic memory or musical novelty; the contrasts between repeat [second] and first presentations refer to melodies heard [musical events] during scanning, i.e., musical episodic memory). Control, healthy control group; PCA, patient group with posterior cortical atrophy; mAD, patient group with memory-led presentation of Alzheimer's disease.

Musical semantic processing (familiar > unfamiliar melody conditions) was associated in the healthy control group with significant activation of bilateral supplementary motor and anterior superior temporal cortices and right inferior frontal gyrus; and in the mAD group, with significant activation of bilateral supplementary motor cortex and left anterior superior temporal cortex. No significant activation for musical semantic processing was identified in the PCA group. Musical novelty processing (unfamiliar > familiar melody conditions) was associated in the healthy control group with activation of right precuneus; there were no significant activations within either patient group for this contrast. Comparing participant groups on the musical semantic memory contrasts revealed a significant activation difference (Fig. 3) between the healthy control group and the mAD group in right inferior frontal gyrus. From inspection of plots of effect size (Fig. 3), this interaction was driven chiefly by reduced activation to familiar melodies in the mAD group. There were no other significant activation differences between participant groups at the prescribed threshold.

Incidental musical episodic memory (repeat > first presentation of repeated melodies) and musical encoding (first > repeat presentation of repeated melodies) revealed no significant activations within any participant group at *p* < .05_FWE_ within the pre-specified anatomical small volume of interest. However, comparing groups revealed significant differences between the healthy control group and each of the patient groups for musical episodic memory processing: for the comparison between healthy control and mAD groups, this activation difference occurred in left precuneus while for the comparison between healthy control and PCA groups the activation difference occurred in right posterior cingulate cortex (Fig. 3). From inspection of plots of effect size, the interaction versus healthy controls was driven for the mAD group chiefly by abnormal activation of precuneus (relative to baseline) during melody encoding; and for the PCA group, by abnormal activation of posterior cingulate cortex by repeated melodies. There were no significant activation differences between patient groups at the prescribed threshold.

In a regression analysis of out-of-scanner behavioural performance (d-prime) against peak effect size in the relevant anatomical regions, no significant correlations with output behaviour were found for the patient cohort for either the musical semantic memory or musical episodic memory contrasts (all *p* > .05).

## 4 Discussion

We have demonstrated a functional neuroanatomical basis for alterations of musical memory in mAD and its major ‘visual’ variant syndrome, PCA. In line with our prior experimental predictions, relative to the healthy control group both syndromic groups showed altered activation of postero-medial cortical regions during incidental episodic processing of repeated melodies and the mAD group additionally showed altered activation of inferior frontal cortex during semantic processing of familiar (relative to unfamiliar) melodies. Out-of-scanner behavioural testing demonstrated the anticipated neuropsychological profiles of impaired musical episodic memory but retained musical semantic memory in both patient groups: however, behavioural performance in the patient groups did not correlate with brain activation profiles, suggesting these profiles may represent true disease signatures rather than simply compensatory effects.

During processing of familiar melodies, both the reference healthy control group and the mAD group showed activation of a predominantly anterior fronto-temporal cortical network previously implicated in musical semantic memory in healthy brain (Jacobsen et al., 2015, Platel et al., 2003, Groussard, La Joie et al., 2010, Groussard, Rauchs et al., 2010, Sikka et al., 2015) (Fig. 2). Involvement of anterior temporal cortex is consistent with the key integrative function of this region in other domains of semantic knowledge and previous evidence that anterior temporal degeneration is associated with impaired recognition of familiar music (Hsieh et al., 2011, Golden, Downey et al., 2015). Inferior and dorso-medial prefrontal regions may be engaged in anticipating syntactical structure in familiar music and implicitly preparing motor responses (Demorest et al., 2010, Pereira et al., 2011). The healthy control group showed activation of right precuneus by previously unfamiliar melodies: this response was lost in the patient groups, consistent with the targeting by AD pathology of hippocampus and linked temporo-parietal circuits that decode musical novelty (Herdener et al., 2010, Demorest et al., 2010, Kafkas and Montaldi, 2014). The lack of a significant difference between patient and control groups on the musical novelty contrast may simply reflect the relatively small cohort size here; alternatively, it may imply that the neuroanatomical substrates of novelty processing within the distributed functional network vary widely between individuals, which could plausibly reflect the complex behavioural and experiential influences that modulate the novelty value of particular stimuli [i.e., musical novelty is not simply the cognitive ‘mirror image’ of familiarity (Kafkas & Montaldi, 2014)]. Although inferior frontal cortex is not a canonical site of pathological involvement in AD and did not emerge as a site of significant disease-related atrophy in the present AD cohort (Fig. 1), reduced activation of this region by familiar melodies in the mAD group relative to the healthy control group here might reflect an abnormal interaction of distributed fronto-temporo-parietal brain networks that decode novelty and familiarity (Kafkas & Montaldi, 2014). In contrast, dorsal medial prefrontal cortex did not emerge as a functional locus of altered musical semantic processing in either AD syndromic group: as proposed by Jacobsen and colleagues (Jacobsen et al., 2015), this region may be relatively resistant to the effects of AD pathology and may therefore provide a substrate for relative preservation of musical semantic memory in AD.

We did not identify any within-group functional neuroanatomical correlates of incidental episodic melody processing at the prescribed threshold. This is in line with previous work employing a similar paradigm in the healthy brain (Jacobsen et al., 2015); melody repetition is likely to be intrinsically less salient than prior familiarity, particularly where (as here) there is no task demanding active recall or recognition during scanning. Nevertheless, incidental processing of repeated melodies left its traces in the comparison between patients and healthy controls (Fig. 3). The mAD group failed to deactivate precuneus normally on first presentation of melodies (implicit melody encoding) while the PCA group showed abnormally increased activation of posterior cingulate cortex on second presentation of melodies (implicit melody recollection). Postero-medial cortex constitutes a principal projection zone of the hippocampal outflow (Leech, Kamourieh, Beckmann & Sharp, 2011) and a core target of AD pathology, here as in previous studies (Lehmann et al., 2010) (Fig. 1). However, the paradoxical over-activation of this region in patients relative to healthy controls argues against any simple effect of signal attenuation by atrophy. While information for melodies remains limited, our findings fit with previous fMRI evidence for the processing of other kinds of memoranda by postero-medial cortex. Task-induced deactivation of postero-medial cortex has been shown to predict successful memory formation in both younger and healthy older cohorts (Cavanna and Trimble, 2006, Daselaar et al., 2004), whereas memory impairment in AD has been linked to impaired deactivation and paradoxical activation of postero-medial cortex (Celone et al., 2006, Lustig et al., 2003, Rombouts et al., 2005Celone et al., 2006; Lustig et al., 2003; Rombouts, Barkhof, Goekoop, Stam & Scheltens, 2005).

The precise functions of the subregions composing postero-medial cortex and the impact of different AD phenotypes on these subregions continue to be defined. However, the syndromic profiles for incidental musical episodic memory observed here are consistent with current formulations derived from extra-musical cognitive domains. Behaviourally, mAD is associated with impaired memory encoding while PCA has been found to be particularly associated with impaired memory retrieval (Tsai, Teng, Liu & Mendez, 2011Tsai, Teng, Liu & Mendez, 2011); this would predict relatively greater disruption of melody encoding (first presentation of melodies) in mAD and relatively greater disruption of melody retrieval (second presentation of melodies) in PCA, as indexed by condition-specific activation profiles here (Fig. 3). In mAD, dysfunction of precuneus might impair the preparation of responses to external sensory events and encoding of those events into memory (Cavanna & Trimble, 2006); while in PCA, dysfunction of posterior cingulate cortex might underpin impaired attentional shifts across internal states (for example, during reanimation of memories) as well as the external sensory environment (Leech & Sharp, 2014), (Shakespeare, Pertzov, Yong, Nicholas & Crutch, 2015). We do not, of course, argue that these putative mechanisms or the neuroanatomical correlates observed here are mutually exclusive or syndrome-specific: indeed, the correlation between musical semantic and episodic memory performance in the present patient cohort argues for at least some functional inter-dependence of these two musical memory systems. The basis for this putative inter-dependence has not been defined but could, for example, reflect a joint effect from affective valence or emotion coding in both memory systems (El Haj et al., 2012, Pereira et al., 2011). Emotion processing is more salient for music than for many other memoranda and could plausibly ‘bind’ episodic and identity information conveyed by melodies. The extent of episodic – semantic interaction and the overall balance of effects observed is likely to depend on the particular musical memory paradigm employed.

Our findings substantiate previous evidence that musical memory in AD is not a unitary phenomenon: in the present patient cohort, deficits of incidental musical episodic memory led deficits of musical semantic memory both in mAD and PCA. This differential vulnerability of musical memory is underpinned by separable functional neuroanatomical substrates: the core neuroanatomical substrate for altered musical episodic processing in both AD and PCA lies within the postero-medial cortical ‘hub’ zone of the core ‘default-mode’ network targeted by AD pathology. On the other hand, the key functional neural substrate of musical semantic memory lies in prefrontal cortex and the impact of different AD phenotypes on this anterior substrate appears to be more variable. Taken together, these findings suggest considerable scope for heterogeneity of musical memory deficits both between memory systems and between individual patients with AD (Baird and Samson, 2015, Cuddy et al., 2015, Groussard et al., 2013, Jacobsen et al., 2015, Omar et al., 2010, Omar et al., 2012, Vanstone et al., 2012). The neuroanatomical profiles identified here and the complexity of functional alterations (in particular, abnormal increased activations) produced by AD relative to the healthy brain underline the potential of music to capture dynamic disease-associated effects in vulnerable neural networks. The present findings add to a coherent body of evidence suggesting that disordered analysis of complex auditory environments is a robust and relevant functional marker of AD pathology.

This study has several limitations and raises important issues for future work. Most fundamentally, our simple, task-free fMRI paradigm deliberately eschewed much of the complexity of musical semantic and episodic memory. The memoranda coded by each of these systems are likely to be hierarchical and multi-componential: explicit melody recognition and recall of musical episodes often entail the activation of associated knowledge about musical objects and associated detail about musical events. If indeed (as our data suggest) musical semantic and episodic memory interact, this could (for example) differentially affect encoding and ‘novelty’ processing at first presentation of previously familiar versus previously unfamiliar melodies, an effect we did not explore here. The interactions of both memory systems with emotion processing mechanisms warrant further investigation, given the relevance of emotional response to music listening under many circumstances. Futhermore, the post-scan behavioural tests used here to provide indices of episodic and semantic musical memory performance did not strictly mirror the ‘incidental’ musical memory engendered during scanning. Future studies should begin to disentangle this complexity. In particular, the use of explicit in-scanner behavioural tasks will allow more direct interpretation of the observed functional neuroanatomical effects, though care will be needed to adjust for task difficulty and performance monitoring factors.

The present study population raises further caveats. The PCA group here was relatively small; our findings should be further corroborated in larger cohorts covering the full phenotypic spectrum of AD, including the logopenic and frontal variants to more comprehensively assess the role of peri-Sylvian and frontal cortices in musical memory in AD. It will be important to assess the wider population of AD and in particular the more frequent scenario of older age onset, ultimately with histopathological correlation. Whilst individuals with younger onset AD and late onset AD all show atrophy affecting the core default mode network, involvement of precuneus tends to be relatively more prominent in those with young onset disease (Möller et al., 2013). Such regional variations in disease pattern suggest that our findings should be extrapolated with caution to individuals with more typical, later onset AD. There is clearly a need to include older patients in future studies of musical memory in AD, while calibrating for the effects of cerebrovascular disease and other comorbidities in that age group. It remains unclear how musical memory evolves over the clinical course of AD: this will only be established by longitudinal study of episodic and semantic musical memory in AD including individuals with mild cognitive impairment and biomarker evidence of underlying AD and ideally, presymptomatic carriers of pathogenic mutations. It is likely that profiles of musical memory (including the relative prominence of episodic and semantic deficits) differ between AD and other neurodegenerative proteinopathies, such as the frontotemporal dementias (Omar et al., 2010, Omar et al., 2012): behavioural and neuroanatomical correlates of musical memory in these diseases should be compared directly and assessed in relation to other components of music cognition (Golden et al., 2017, Omar et al., 2010, Omar et al., 2012). The roles played by particular components of the musical memory networks implicated here will only be fully elucidated by paradigms that incorporate techniques with high temporal resolution (such as magnetoencephalography) that can track dynamic connectivity shifts between memory phases and among brain regions.

Few standard, currently available neuropsychological paradigms rival the propensity of music to engage emotional and social signal processing mechanisms while also potentially reanimating memories. Improved understanding of musical memory in AD should in turn inform rational music-based therapies. Music already provides a welcome source of comfort for patients and their caregivers. However, music-based therapies may have cognitive benefits beyond enjoyment and improved quality of life. For example, the use of personalised playlists of familiar music may help people living with dementia to access specific (and especially, emotionally salient) autobiographical memories (El Haj et al., 2012, Särkamö et al., 2014). The present work provides a neurobiological rationale for such strategies in AD, analogous to functional neuroanatomical studies of musical neurorehabilitation following stroke (Sarkamo & Sihvonen, 2018). We hope that work of this kind will guide the development of such therapies, by suggesting fruitful targets within the domain of music cognition and providing surrogate therapeutic markers of altered brain function in Alzheimer's and other neurodegenerative diseases.

## 5 Conclusions

Our findings demonstrate a functional neuroanatomical basis for the alterations in and differential vulnerability of musical semantic and incidental episodic memory in mAD and its major ‘visual’ variant syndrome, PCA. We propose musical memory as a window to probe neural network changes in Alzheimer's disease, and a model system to inform the development of future musical interventions for people living with dementia.

## Ethics approval and consent to participate

The study was approved by the local institutional research ethics committee (London Queen Square, 13/LO/0005) and conducted in accordance with Declaration of Helsinki guidelines. All patients and healthy individuals gave written informed consent to participate.

## Consent for publication

All participants and healthy individuals gave written informed consent for publication.

## Competing interests

The authors report no relevant competing interests.

## Funding

The Dementia Research Centre is supported by Alzheimer's Research UK, the Brain Research Trust and the Wolfson Foundation. This work was funded by the Alzheimer's Society, the Wellcome Trust, the UK Medical Research Council and the NIHR UCLH Biomedical Research Centre. The funding bodies did not contribute to the design of the study, the collection analysis or interpretation of data, or in writing the manuscript.
