# Supplementary material for: The functional neuroanatomy of musical memory in Alzheimer's disease
Source: Cortex. 2019 Jun;115:357–70. doi: 10.1016/j.cortex.2019.02.003 (PMC6525150; doi:10.1016/j.cortex.2019.02.003)
Supplement: Multimedia component 1 [file mmc1.docx]

**Supplementary Material: The functional neuroanatomy of musical memory in Alzheimer’s disease, by CF Slattery et al**


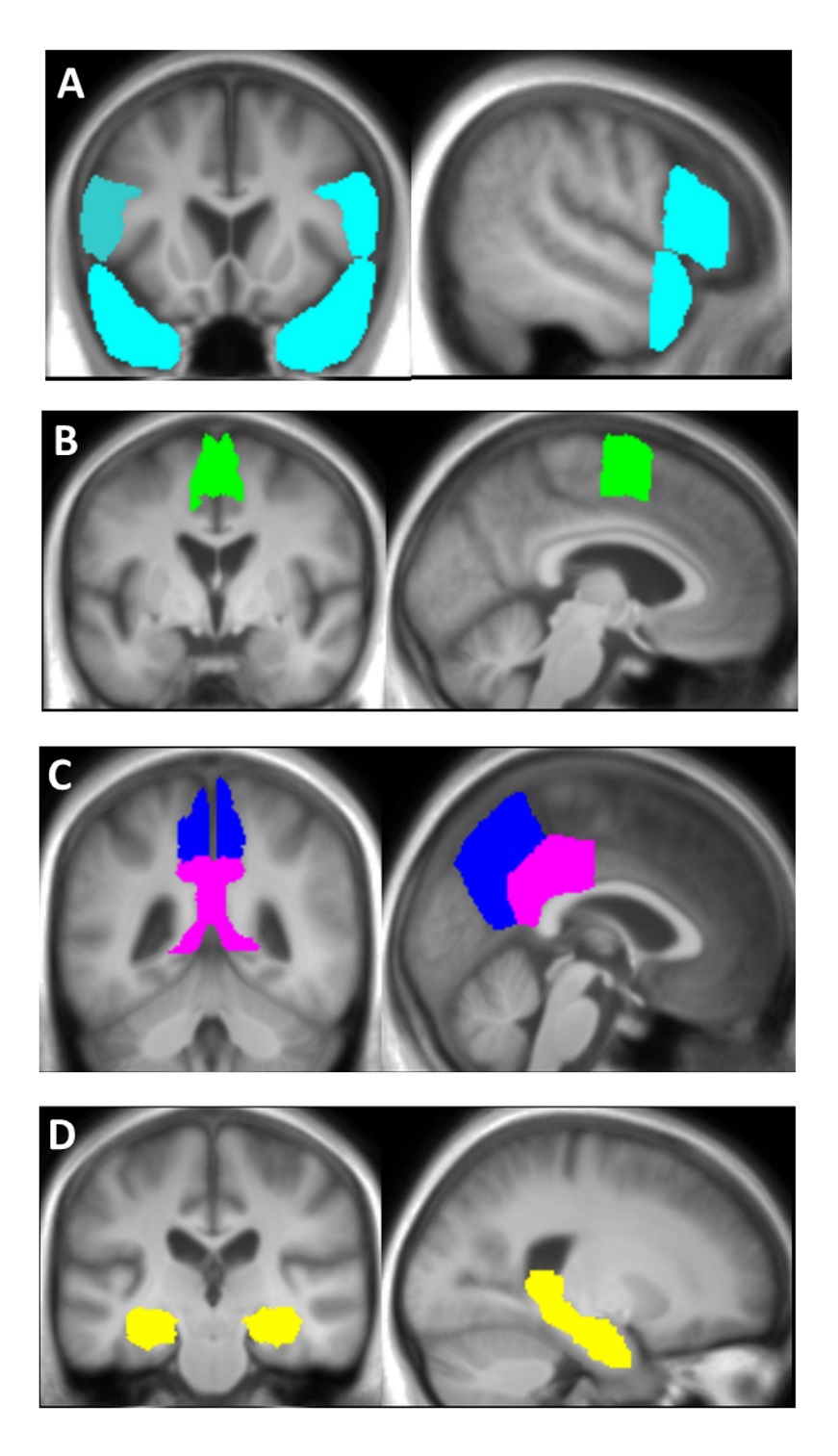


**Figure S1.** Representative coronal (left) and sagittal (right) sections of the anatomically-defined small volumes used in the fMRI analysis are shown, projected on the study-specific group mean T1-weighted structural MR brain image. Anatomical regions were derived from the Harvard-Oxford Brain Atlas [36] and created in FSL view[[37](#_ENREF_37)], as follows: **A**, inferior frontal gyrus, frontal operculum and anterior temporal cortex (cyan); **B**, supplementary motor cortex (green); **C**, posterior cingulate cortex (magenta) and precuneus (blue); **D**, hippocampi (yellow). Volumes shown in **A** and **B** were used in analysis of the contrast assessing musical semantic memory; volumes shown in **C** and **D** were used in analysis of contrasts assessing musical episodic memory; and both regions were used in analysis of the contrast assessing musical novelty processing (see text)

**Table S1.** Familiar melodies presented in the fMRI experiment

| **Composer** | **Musical piece** | **Notes** (no.) | **Pitch range**  (notes) | **Pitch change** |
| --- | --- | --- | --- | --- |
|  |  |  |  | (semitones)* |
| Bach | Fugue in D minor | 32 | A0 - A1 | 7.07 |
| Bach | Jesu Joy of Man’s Desiring | 32 | D1 - G2 | 2.87 |
| Bach | Minuet in G: excerpt 1 | 32 | A1 - E2 | 1.67 |
| Bach | Minuet in G: excerpt 2 | 32 | F1# - G2 | 3.91 |
| Bach | Toccata in D minor | 32 | F1 - A2# | 2.58 |
| Barber | Adagio for Strings | 16 | A1 - D2# | 1.84 |
| Beethoven | Fur Elise: excerpt 1 | 32 | E0 - E3 | 8.44 |
| Beethoven | Fur Elise: excerpt 2 | 32 | E0 - E2 | 6.74 |
| Beethoven | Moonlight Sonata | 32 | G0# -F1# | 5.93 |
| Beethoven | Ode to Joy | 32 | D1 - E2 | 3.67 |
| Bizet | Toreador’s Song (Carmen) | 18 | C1 - D2 | 3.18 |
| Bizet | Habanera (Carmen): excerpt 1 | 35 | D1 - C2# | 2.40 |
| Bizet | Habanera (Carmen): excerpt 2 | 29 | D1 - D2 | 2.23 |
| Boccherini | Minuet (String Quintet in E) | 30 | D1 - A2 | 4.12 |
| Brahms | Hungarian Dance No 5 | 19 | F1# - A2 | 3.27 |
| Charpentier | Prelude (Te Deum) | 21 | G1 - G2 | 2.87 |
| Delibes | Mazurka (Coppelia) | 21 | G1 - C3 | 4.61 |
| Delibes | Flower Duet (Lakme) | 32 | G1 - D2# | 1.74 |
| Dvorak | Humoreske: excerpt 1 | 21 | G1 - B2 | 3.35 |
| Dvorak | Humoreske: excerpt 2 | 28 | C1 - A2 | 2.70 |
| Dvorak | New World Symphony, Adagio | 16 | F1 - C2 | 2.42 |
| Grieg | Morning Mood (Peer Gynt): excerpt 1 | 32 | F1 - D2 | 2.95 |
| Grieg | Morning Mood (Peer Gynt): excerpt 2 | 32 | E1 - C2# | 3.43 |
| Grieg | Hall of the Mountain King (Peer Gynt) excerpt 1 | 32 | C1# - A1 | 3.03 |
| Grieg | Hall of the Mountain King (Peer Gynt) excerpt 2 | 32 | B0 - B1 | 3.60 |
| Handel | Hornpipe (Water Music) | 28 | C1 - G1 | 2.87 |
| Handel | Arrival of Queen of Sheba: excerpt 1) | 32 | B0 - E2 | 2.35 |
| Handel | Arrival of Queen of Sheba: excerpt 2 | 32 | G1 - G2 | 3.79 |
| Joplin | The Entertainer: excerpt 1 | 36 | D1 - E2 | 5.14 |
| Joplin | The Entertainer: excerpt 2 | 48 | D1 - E2 | 2.6 |
| Mozart | Eine Kleine Nachtmusik | 18 | D1 - D2 | 4.25 |
| Mozart | Piano Concerto No 21, Mov 2 | 26 | C2 - D3 | 4.68 |
| Mozart | Symphony No 40, Mov 1: excerpt 1 | 40 | C1 - A1# | 2.86 |
| Mozart | Symphony No 40, Mov 1: excerpt 2 | 31 | C2# - C3 | 2.96 |
| Mozart | Turkish Rondo (Piano Sonata No 11) | 43 | G1# - C3 | 2.35 |
| Offenbach | Infernal Gallop (Orpheus): excerpt 1 | 32 | A1 - D3 | 3.94 |
| Offenbach | Infernal Gallop (Orpheus): excerpt 2 | 32 | G1 - G2 | 3.20 |
| Prokofiev | Peter’s theme (Peter and the Wolf) | 25 | G1 - C3 | 4.28 |
| Puccini | Nessun Dorma | 16 | B1 - G2 | 2.52 |
| Quilter | Upon St Paul’s | 16 | D1 - D2 | 4.72 |
| Ravel | Bolero | 33 | C1 - D2 | 1.99 |
| Saint-Saens | Danse Macabre | 32 | D1 - A1# | 2.26 |
| Strauss | Radetsky March | 43 | C1 - B1 | 3.34 |
| Strauss | Tritsch Tratsch Polka | 38 | B0 - E2 | 5.10 |
| Tchaikovsky | Waltz of the Flowers (Nutcracker) | 27 | F1 - F2 | 4.17 |
| Tchaikovsky | Dance of the Little Swans (Swan Lake) | 32 | F0# -F2# | 4.84 |
| Vivaldi | Spring (The Four Seasons) | 32 | B1 - B2 | 3.03 |
| Wagner | Ride of the Valkyries | 18 | F0# - A1 | 5.02 |
| *Mean:* |  | *29.4* |  | *3.6* |

The 48 familiar melodies used are indicated, with relevant stimulus parameters; all stimuli were edited to duration 8 seconds and presented using a pleasant synthetic timbre with fixed overall (root-mean-square) intensity. Familiar melodies comprised 48 excerpts from tunes widely known among older British people. Popular classical instrumental (non-vocal) tunes with minimal verbal associations were chosen to reduce any effects from verbal labelling. In a stimulus selection pilot study (five healthy British individuals, all >50 years, none of whom participated subsequently in the fMRI study), the tune excerpts selected were classified as highly familiar with ≥80% consensus by the pilot control group. *standard deviation for inter-tone pitch variation across the 8 second excerpt.
